# Foreign body in the bronchus of a child: the importance of making the correct diagnosis

**DOI:** 10.1590/0100-3984.2015.0169

**Published:** 2016

**Authors:** Antonio Gabriel de Jesus Barbosa, Diana Penha, Gláucia Zanetti, Edson Marchiori

**Affiliations:** 1 Universidade Federal do Rio de Janeiro (UFRJ), Rio de Janeiro, RJ, Brazil.; 2 Heart and Chest Hospital NHS Foundation Trust, Liverpool, UK.

Dear Editor,

A 7-year-old female presented to the emergency room with a 24-hour history of dyspnea, fever, and an episode of syncope. Physical examination showed an axillary temperature of 38°C and absent breath sounds on the left. The blood workup showed a leukocyte count of 21,000 cells/mm^3^, with eight rods. Computed tomography (CT) of the chest was performed, after which the patient was admitted with a presumptive diagnosis of pneumonia. On the first day of hospitalization, the patient showed a decrease in oxygen saturation, cyanosis, and cardiopulmonary arrest, all of which were reversed after routine cardiopulmonary resuscitation maneuvers. After 30 days of antibiotic treatment and intensive care, there was improvement in the clinical and biochemical parameters. Nevertheless, on pulmonary auscultation, breath sounds were still diminished on the left. In view of that finding, another CT scan of the chest was performed.

**Figure 1:**
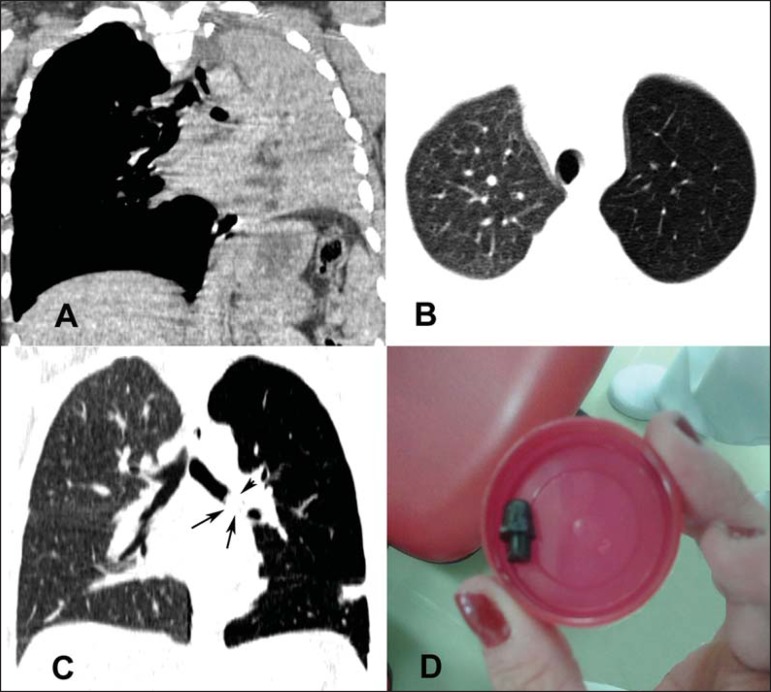
In **A**, CT scan of the chest, showing total atelectasis of the left lung. In **B** and **C**, a second CT scan, acquired one month later, showing a diffuse reduction in attenuation in the left lung, with discrete hyperinflation. Note also, in **C**, the dense image within the left main bronchus (arrows). In **D**, photograph of the foreign body removed by bronchoscopy.

The first chest CT had shown atelectasis in the left lung (Figure 1A), which was erroneously interpreted as pneumonia. The second CT scan, acquired one month after, showed a diffuse reduction in the attenuation of the left lung parenchyma, with hyperinflation (Figures 1B and 1C). The possibility of partial bronchial obstruction was suggested, strengthened by the rounded image observed in the distal third of the left main bronchus, with a diameter of 20 mm. The patient underwent bronchoscopy, and a foreign body (part of a plastic ballpoint pen) was identified and removed (Figure 1D). She was discharged a few days later.

Foreign body aspiration (FBA) is a common event, with significant potential for morbidity and mortality^(1-4)^. Children, particularly males under three years of age, are most often affected^(1,5,6)^.

A diagnosis of FBA is not always easily made, because, in most cases, the parents did not witness the accident and the suspicion must be based on clinical history, physical examination, and complementary diagnostic methods^(1)^. In general, the clinical presentation depends on factors such as the type, size, and location of the foreign body, as well as the age of the patient. It is of note that some victims are asymptomatic and show no alterations on physical examination^(1,5,6)^.

The majority of aspirated foreign bodies are radiolucent, and the image findings on radiographic examinations are, therefore, secondary to their presence in the airway and depend on the size of the foreign body, site of impaction, and degree of obstruction caused^(3,5)^. The main radiological findings are atelectasis, air trapping/pulmonary hyperinflation and identification of a radiopaque foreign body, as well as, less often, pneumomediastinum and pneumothorax^(2)^.

CT is the examination of choice for the evaluation of a large number of lung diseases, as shown in recent studies in the radiology literature of Brazil^(7-12)^. CT and bronchoscopy are useful in the investigation of cases of persistent respiratory symptoms in which the diagnostic hypothesis is FBA. The diagnosis of the FBA should be made early because a delay in its recognition and treatment can result in definitive sequelae or fatal damage. In many cases, recurrent respiratory diseases are treated for weeks or months before there is suspicion of FBA^(1)^.

In conclusion, the presence of acute respiratory symptoms accompanied by atelectasis in children should be a signal for the likelihood of FBA, making it an early indication for bronchoscopy, which can be diagnostic as well as therapeutic.
